# Supplementary material for: Testing Potential Transfer Effects in Heritage and Adult L2 Bilinguals Acquiring a Mini Grammar as an Additional Language: An ERP Approach
Source: Brain Sci. 2022 May 20;12(5):669. doi: 10.3390/brainsci12050669 (PMC9139276; doi:10.3390/brainsci12050669)
Supplement: Supplementary file 1 [file brainsci-12-00669-s001.zip › Supplementary_Materials_S1.pdf]

Table S1. Participant's background information.

Subject	Language Group	Proficiency_German (%)	Proficiency_Italian (%)	Proficiency_English	Sex	AoA	SESM	Age	LoR
**001**	German L2er	97.33	NA	83.75	Female	11	0	26.31	15.31
**002**	German L2er	96	NA	66.25	Female	7	2	18.28	11.28
**003**	German L2er	98.67	NA	76.25	Female	8	4	24.02	16.02
**004**	German L2er	96	NA	75	Female	NA	1	24.82	NA
**005**	German L2er	93.33	NA	83.75	Male	11	1	32.03	21.03
**006**	German L2er	94.67	NA	58.75	Male	10	1	28.05	18.05
**007**	German L2er	96	NA	88.75	Female	12	0	33.75	21.75
**008**	German L2er	97.33	NA	88.75	Male	13	3	29.59	16.59
**009**	German L2er	98.67	NA	76.25	Female	7	1	25.48	18.48
**010**	German L2er	93.33	NA	91.25	Female	6	0	22.01	16.01
**011**	German L2er	96	NA	63.75	Female	7	3	18.57	11.57
**012**	German L2er	98.67	NA	63.75	Female	11	1	26.9	15.9
**013**	German L2er	93.33	NA	61.25	Female	7	1	23.86	16.86
**014**	German L2er	92	NA	82.5	Female	6	1	21.18	15.18
**015**	German L2er	92	NA	61.25	Female	8	1	24.06	16.06
**016**	German L2er	97.33	NA	96.25	Male	9	4	25.98	16.98
**017**	German L2er	92	NA	60	Female	11	1	NA	NA
**018**	German L2er	92	NA	60	Female	10	1	23.81	13.81
**019**	German L2er	70.59	NA	48.75	Male	10	1	25.38	15.38
**020**	German L2er	96	NA	61.25	Male	11	3	21.43	10.43
**021**	German L2er	92	NA	60	Male	11	1	20.6	109.6
**022**	German L2er	98.67	NA	66.25	Female	11	1	26.73	15.73
**023**	German L2er	97.33	NA	76.25	Female	10	1	26.51	16.51
**024**	German L2er	97.33	NA	62.5	Female	10	1	27.42	17.42
**025**	German L2er	94.67	NA	62.5	Male	11	1	28.02	17.02

**026**	German L2er	86.67	NA	55	Male	10	1	26.72	16.72
**027**	German L2er	93.33	NA	60	Female	8	2	22.75	14.75
**028**	German L2er	96	NA	52.5	Female	NA	2	23.38	NA
**029**	Italian HSs	96	92	78.75	Female	0	2	23.57	23.57
**030**	Italian HSs	100	82.67	68.75	Male	2	0	22.61	20.61
**031**	Italian HSs	84	58.67	55	Female	2	2	24.77	22.77
**032**	Italian HSs	100	62.67	66.25	Female	3	0	23.63	20.63
**033**	Italian HSs	92	80	71.25	Female	0	0	19.16	19.16
**034**	Italian HSs	88	76	53.75	Female	3	0	25.18	22.18
**035**	Italian HSs	94.67	72	45	Female	4	0	25.01	21.01
**036**	Italian HSs	89.33	69.33	45	Female	4	0	19.85	15.85
**037**	Italian HSs	98.67	78.67	67.5	Female	NA	0	30.08	30.08
**038**	Italian HSs	90.67	78.67	58.75	Female	0	0	22.84	22.84
**039**	Italian HSs	98.67	85.33	66.25	Female	2	0	23.76	21.76
**040**	Italian HSs	97.33	89.33	52.5	Female	3	0	24.25	21.25
**041**	Italian HSs	96	58.67	58.75	Male	4	0	35.5	31.5
**042**	Italian HSs	92	82.67	80	Male	0	2	22.49	22.49
**043**	Italian HSs	96	65.33	68.75	Female	4	0	21.38	17.38
