# Supplementary material for: Testing Potential Transfer Effects in Heritage and Adult L2 Bilinguals Acquiring a Mini Grammar as an Additional Language: An ERP Approach
Source: Brain Sci. 2022 May 20;12(5):669. doi: 10.3390/brainsci12050669 (PMC9139276; doi:10.3390/brainsci12050669)
Supplement: Supplementary file 1 [file brainsci-12-00669-s001.zip › Supplementary_Materials_S2.pdf]

Table S2. Latin lexical items (showed in their nominative form only) for the L3 Mini-Latin study and their translations in German, Italian and English.

	**Translation**		
	**German**	**Italian**	**English**
**Word Class**			
**Nouns**			
Nupta (f)	Die Braut	La sposa	The bride
Venefica (f)	Die Hexe	La strega	The witch
Vetula (f)	Die alte Frau	La vecchia	The old woman
Ludia (f)	Die Tänzerin	La ballerina	The dancer
Antistita (f)	Die Pfarrerin	La sacerdotessa	The priestess
Puella (f)	Das Mädchen	La banbina	The girl
Hariola (f)	Die Hellseherin	La chiaroveggente	The clairvoyant
Erus (m)	Der Mann	Il signore	The man
Tignarius (m)	Der Schreiner	Il falegname	The carpenter
Magirus (m)	Der Koch	Il cuoco	The cook
Famulus (m)	der Sklave	Lo schiavo	The slave
Pecuarius (m)	der Viehzüchter	L'allevatore	The breeder
Abietarius (m)	der Zimmermann	Il cacrpentiere	The carpenter
Lanius (m)	der Metzger	Il maccellaio	The butcher
**Verbs**			
Siuare	Helfen	Aiutare	To helo
Uocare	Rufen	Chiamare	To call
Rogare	Befragen	Interrogare	To interrogate
Sauciare	Verletzen	Ferire	To hurt
Cruciare	Belästigen	Tormentare	To harass
Necare	Umbringen	Uccidere	To kill

Obiurgare	Tadeln	Rimproverare	To scold
Collustrare	Betrachten	Osservare	To observe
**Adjectives**			
Calicus	Italienisch	Italiano	Italian
Mallus	Französisch	Francese	French
Sercus	Spanisch	Spagnolo	Spanish
Teunus	deutsch	Tedesco	German
Micanus	Britisch	Inglese	English
Tidonus	Amerikanisch	Americano	American
