# Supplementary material for: Testing Potential Transfer Effects in Heritage and Adult L2 Bilinguals Acquiring a Mini Grammar as an Additional Language: An ERP Approach
Source: Brain Sci. 2022 May 20;12(5):669. doi: 10.3390/brainsci12050669 (PMC9139276; doi:10.3390/brainsci12050669)
Supplement: Supplementary file 1 [file brainsci-12-00669-s001.zip › Supplementary_Materials_S3-S26.pdf]

Table S3. Model output for omnibus ANOVA in the HSs, adjective violation 200–500 ms: lateral electrodes.

Effect	DFn	DFd	F	p	η2
(Intercept)	1	14	11.57	0.004	0.30
Condition	1	14	1.58	0.229	0.02
Hemisphere	1	14	6.28	0.025	0.02
Caudality	2	28	5.68	0.008	0.06
Condition:Hemisphere	1	14	1.72	0.211	0.00
Condition:Caudality	2	28	2.57	0.094	0.00
Hemisphere:Caudality	2	28	5.56	0.009	0.01
Condition:Hemisphere:Caudality	2	28	1.36	0.273	0.00

Table S4. Model output for omnibus ANOVA in the HSs, adjective violation 200–500 ms: midline electrodes.

Effect	DFn	DFd	F	p	η2
(Intercept)	1	14	7.57	0.016	0.24
Condition	1	14	0.48	0.500	0.00
Caudality	2	28	10.50	0.000	0.10
Condition:Caudality	2	28	2.14	0.136	0.00

Table S5. Model output for omnibus ANOVA in the HSs, Case violation 200–500 ms: lateral electrodes.

**Effect**	**DFn**	**DFd**	**F**	**p**	**η2**
(Intercept)	1	14	5.07	0.041	0.18
Condition	1	14	18.71	0.001	0.11
Hemisphere	1	14	7.22	0.018	0.02
Caudality	2	28	5.53	0.009	0.05
Condition:Hemisphere	1	14	1.64	0.221	0.00
Condition:Caudality	2	28	1.45	0.251	0.00
Hemisphere:Caudality	2	28	8.14	0.002	0.01
Condition:Hemisphere:Caudality	2	28	0.99	0.385	0.00

Table S6. Model output for omnibus ANOVA in the HSs, Case violation 200–500 ms: midline electrodes.

**Effect**	**DFn**	**DFd**	**F**	**p**	**η2**
(Intercept)	1	14	2.85	0.113	0.12
Condition	1	14	13.46	0.003	0.07
Caudality	2	28	7.78	0.002	0.08
Condition:Caudality	2	28	0.30	0.745	0.00

Table S7. Model output for omnibus ANOVA in the HSs, adjective violation 300–600 ms: lateral electrodes.

**Effect**	**DFn**	**DFd**	**F**	**p**	**η2**
(Intercept)	1	14	3.86	0.070	0.13
Condition	1	14	1.28	0.278	0.02
Hemisphere	1	14	6.93	0.020	0.01
Caudality	2	28	7.38	0.003	0.07
Condition:Hemisphere	1	14	1.55	0.233	0.00
Condition:Caudality	2	28	1.53	0.235	0.00
Hemisphere:Caudality	2	28	6.04	0.007	0.01
Condition:Hemisphere:Caudality	2	28	0.23	0.795	0.00

Table S8. Model output for omnibus ANOVA in the HSs, adjective violation 300–600 ms: midline electrodes.

**Effect**	**DFn**	**DFd**	**F**	**p**	**η2**
(Intercept)	1	14	2.36	0.147	0.09
Condition	1	14	0.38	0.548	0.00
Caudality	2	28	12.29	0.000	0.12
Condition:Caudality	2	28	0.86	0.436	0.00

Table S9. Model output for omnibus ANOVA in the HSs, Case violation 300–600 ms: lateral electrodes.

Effect	DFn	DFd	F	p	η2
(Intercept)	1	14	1.59	0.228	0.07
Condition	1	14	14.20	0.002	0.08
Hemisphere	1	14	8.33	0.012	0.02
Caudality	2	28	8.38	0.001	0.07
Condition:Hemisphere	1	14	0.89	0.363	0.00
Condition:Caudality	2	28	0.45	0.642	0.00
Hemisphere:Caudality	2	28	7.03	0.003	0.01
Condition:Hemisphere:Caudality	2	28	1.43	0.256	0.00

Table S10. Model output for omnibus ANOVA in the HSs, Case violation 300–600 ms: midline electrodes.

Effect	DFn	DFd	F	p	η2
(Intercept)	1	14	0.81	0.383	0.04
Condition	1	14	6.87	0.020	0.04
Caudality	2	28	11.29	0.000	0.09
Condition:Caudality	2	28	0.36	0.703	0.00

Table S11. Model output for omnibus ANOVA in the HSs, adjective violation 600–900 ms: lateral electrodes.

Effect	DFn	DFd	F	p	η2
(Intercept)	1	14	0.95	0.347	0.03
Condition	1	14	1.12	0.308	0.02
Hemisphere	1	14	8.93	0.010	0.01
Caudality	2	28	2.71	0.084	0.03
Condition:Hemisphere	1	14	1.58	0.229	0.00
Condition:Caudality	2	28	1.25	0.302	0.00
Hemisphere:Caudality	2	28	7.87	0.002	0.01
Condition:Hemisphere:Caudality	2	28	0.02	0.979	0.00

Table S12. Model output for omnibus ANOVA in the HSs, adjective violation 600–900 ms: midline electrodes.

Effect	DFn	DFd	F	p	η2
(Intercept)	1	14	0.41	0.530	0.02
Condition	1	14	0.28	0.603	0.00
Caudality	2	28	6.82	0.004	0.09
Condition:Caudality	2	28	0.40	0.676	0.00

Table S13. Model output for omnibus ANOVA in the HSs, Case violation 600–900 ms: lateral electrodes.

**Effect**	**DFn**	**DFd**	**F**	**p**	**η2**
(Intercept)	1	14	1.19	0.293	0.05
Condition	1	14	1.10	0.312	0.01
Hemisphere	1	14	10.07	0.007	0.01
Caudality	2	28	2.73	0.082	0.02
Condition:Hemisphere	1	14	0.99	0.336	0.00
Condition:Caudality	2	28	0.13	0.880	0.00
Hemisphere:Caudality	2	28	5.48	0.010	0.00
Condition:Hemisphere:Caudality	2	28	5.08	0.013	0.00

Table S14. Model output for omnibus ANOVA in the HSs, Case violation 600–900 ms: midline electrodes.

**Effect**	**DFn**	**DFd**	**F**	**p**	**η2**
(Intercept)	1	14	0.83	0.378	0.03
Condition	1	14	0.01	0.916	0.00
Caudality	2	28	6.84	0.004	0.06
Condition:Caudality	2	28	0.84	0.442	0.00

Table S15. Model output for omnibus ANOVA in the German L2ers, adjective violation 200–500 ms: lateral electrodes.

**Effect**	**DFn**	**DFd**	**F**	**p**	**η2**
(Intercept)	1	26	34.93	0.000	0.42
Condition	1	26	6.45	0.017	0.03
Hemisphere	1	26	10.47	0.003	0.03
Caudality	2	52	7.05	0.002	0.04
Condition:Hemisphere	1	26	1.08	0.309	0.00
Condition:Caudality	2	52	1.00	0.374	0.00
Hemisphere:Caudality	2	52	10.47	0.000	0.01
Condition:Hemisphere:Caudality	2	52	0.98	0.382	0.00

Table S16. Model output for omnibus ANOVA in the German L2ers, adjective violation 200–500 ms: midline electrodes.

**Effect**	**DFn**	**DFd**	**F**	**p**	**η2**
(Intercept)	1	26	22.71	0.000	0.33
Condition	1	26	6.05	0.021	0.03
Caudality	2	52	14.28	0.000	0.09
Condition:Caudality	2	52	0.53	0.592	0.00

Table S17. Model output for omnibus ANOVA in the German L2ers, Case violation 200–500 ms: lateral electrodes.

**Effect**	**DFn**	**DFd**	**F**	**p**	**η2**
(Intercept)	1	26	23.28	0.000	0.32
Condition	1	26	0.29	0.594	0.00
Hemisphere	1	26	10.57	0.003	0.03
Caudality	2	52	6.97	0.002	0.03
Condition:Hemisphere	1	26	2.42	0.132	0.00
Condition:Caudality	2	52	2.73	0.074	0.00
Hemisphere:Caudality	2	52	14.94	0.000	0.01
Condition:Hemisphere:Caudality	2	52	1.96	0.151	0.00

Table S18. Model output for omnibus ANOVA in the German L2ers, Case violation 200–500 ms: midline electrodes.

**Effect**	**DFn**	**DFd**	**F**	**p**	**η2**
(Intercept)	1	26	13.17	0.001	0.21
Condition	1	26	0.34	0.567	0.00
Caudality	2	52	14.32	0.000	0.10
Condition:Caudality	2	52	2.56	0.087	0.00

Table S19. Model output for omnibus ANOVA in the German L2ers, adjective violation 300–600 ms: lateral electrodes.

Effect	DFn	DFd	F	p	η2
(Intercept)	1	26	15.67	0.001	0.24
Condition	1	26	7.57	0.011	0.03
Hemisphere	1	26	18.74	0.000	0.03
Caudality	2	52	18.39	0.000	0.10
Condition:Hemisphere	1	26	0.55	0.464	0.00
Condition:Caudality	2	52	1.20	0.310	0.00
Hemisphere:Caudality	2	52	10.27	0.000	0.01
Condition:Hemisphere:Caudality	2	52	1.17	0.317	0.00

Table S20. Model output for omnibus ANOVA in the German L2ers, adjective violation 300–600 ms: midline electrodes.

Effect	DFn	DFd	F	p	η2
(Intercept)	1	26	5.45	0.028	0.10
Condition	1	26	6.03	0.021	0.03
Caudality	2	52	29.03	0.000	0.16
Condition:Caudality	2	52	0.47	0.629	0.00

Table S21. Model output for omnibus ANOVA in the German L2ers, Case violation 300–600 ms: lateral electrodes.

**Effect**	**DFn**	**DFd**	**F**	**p**	**η2**
(Intercept)	1	26	6.37	0.018	0.11
Condition	1	26	0.56	0.461	0.00
Hemisphere	1	26	21.12	0.000	0.03
Caudality	2	52	18.15	0.000	0.10
Condition:Hemisphere	1	26	1.15	0.294	0.00
Condition:Caudality	2	52	2.62	0.083	0.00
Hemisphere:Caudality	2	52	14.66	0.000	0.01
Condition:Hemisphere:Caudality	2	52	2.17	0.124	0.00

Table S22. Model output for omnibus ANOVA in the German L2ers, Case violation 300–600 ms: midline electrodes.

**Effect**	**DFn**	**DFd**	**F**	**p**	**η2**
(Intercept)	1	26	0.74	0.399	0.01
Condition	1	26	0.63	0.433	0.00
Caudality	2	52	26.62	0.000	0.16
Condition:Caudality	2	52	2.99	0.059	0.00

Table S23. Model output for omnibus ANOVA in the German L2ers, adjective violation 600–900 ms: lateral electrodes.

**Effect**	**DFn**	**DFd**	**F**	**p**	**η2**
(Intercept)	1	26	10.07	0.004	0.18
Condition	1	26	4.05	0.055	0.02
Hemisphere	1	26	20.08	0.000	0.02
Caudality	2	52	7.98	0.001	0.05
Condition:Hemisphere	1	26	0.87	0.361	0.00
Condition:Caudality	2	52	1.16	0.321	0.00
Hemisphere:Caudality	2	52	5.89	0.005	0.00
Condition:Hemisphere:Caudality	2	52	1.25	0.294	0.00

Table S24. Model output for omnibus ANOVA in the German L2ers, adjective violation 600–900 ms: midline electrodes.

**Effect**	**DFn**	**DFd**	**F**	**p**	**η2**
(Intercept)	1	26	3.65	0.067	0.07
Condition	1	26	3.47	0.074	0.02
Caudality	2	52	15.31	0.000	0.11
Condition:Caudality	2	52	0.41	0.663	0.00

Table S25. Model output for omnibus ANOVA in the German L2ers, Case violation 600–900 ms: lateral electrodes.

**Effect**	**DFn**	**DFd**	**F**	**p**	**η2**
(Intercept)	1	26	5.65	0.025	0.11
Condition	1	26	0.09	0.764	0.00
Hemisphere	1	26	16.83	0.000	0.02
Caudality	2	52	6.27	0.004	0.03
Condition:Hemisphere	1	26	3.45	0.075	0.00
Condition:Caudality	2	52	4.07	0.023	0.00
Hemisphere:Caudality	2	52	5.01	0.010	0.00
Condition:Hemisphere:Caudality	2	52	2.20	0.121	0.00

Table S26. Model output for omnibus ANOVA in the German L2ers, Case violation 600–900 ms: midline electrodes.

**Effect**	**DFn**	**DFd**	**F**	**p**	**η2**
(Intercept)	1	26	1.62	0.214	0.03
Condition	1	26	0.27	0.607	0.00
Caudality	2	52	13.24	0.000	0.09
Condition:Caudality	2	52	6.97	0.002	0.01
